# Supplementary material for: The unequal effects of the health–economy trade-off during the COVID-19 pandemic
Source: Nat Hum Behav. 2023 Nov 16;8(2):264–75. doi: 10.1038/s41562-023-01747-x (PMC10896714; doi:10.1038/s41562-023-01747-x)
Supplement: Supplementary file 2 — Reporting Summary [file 41562_2023_1747_MOESM2_ESM.pdf]

nature portfolio

Corresponding author(s): Marco Pangallo
Last updated by author(s): Sep 3, 2023

Reporting Summary

Nature Portfolio wishes to improve the reproducibility of the work that we publish. This form provides structure for consistency and transparency in reporting. For further information on Nature Portfolio policies, see our Editorial Policies and the Editorial Policy Checklist.

Statistics

For all statistical analyses, confirm that the following items are present in the figure legend, table legend, main text, or Methods section.

n/a	Confirmed	
☐	☒	The exact sample size (*n*) for each experimental group/condition, given as a discrete number and unit of measurement
☒	☐	A statement on whether measurements were taken from distinct samples or whether the same sample was measured repeatedly
☐	☒	The statistical test(s) used AND whether they are one- or two-sided<br>*Only common tests should be described solely by name; describe more complex techniques in the Methods section.*
☒	☐	A description of all covariates tested
☒	☐	A description of any assumptions or corrections, such as tests of normality and adjustment for multiple comparisons
☐	☒	A full description of the statistical parameters including central tendency (e.g. means) or other basic estimates (e.g. regression coefficient) AND variation (e.g. standard deviation) or associated estimates of uncertainty (e.g. confidence intervals)
☐	☒	For null hypothesis testing, the test statistic (e.g. *F*, *t*, *r*) with confidence intervals, effect sizes, degrees of freedom and *P* value noted<br>*Give P values as exact values whenever suitable.*
☐	☒	For Bayesian analysis, information on the choice of priors and Markov chain Monte Carlo settings
☒	☐	For hierarchical and complex designs, identification of the appropriate level for tests and full reporting of outcomes
☒	☐	Estimates of effect sizes (e.g. Cohen's *d*, Pearson's *r*), indicating how they were calculated

Our web collection on statistics for biologists contains articles on many of the points above.

Software and code

Policy information about availability of computer code

Data collection	The data for both the economic and epidemic modules have been collected using standard software packages (Python and R). Census data have been obtained through the ACS API: https://cran.r-project.org/web/packages/acs/index.html.<br>We used Python 3.8.0, and the following versions of the Python packages: numpy==1.20.3; pandas==1.3.5; openpyxl==3.1.2.<br>We used R 4.3.1, and the following versions of the R packages: data.table_1.14.8, cowplot_1.1.1, tidycensus_1.4.4, latex2exp_0.9.6, gridExtra_2.3, zoo_1.8-12, stringr_1.4.0, ggrepel_0.9.1, ggplot2_3.3.5, reshape2_1.4.4, readxl_1.3.1, dplyr_1.0.7, sf_1.0-14
Data analysis	The data for both the economic and epidemic modules have been analyzed using standard software packages (Python and R)<br>We used Python 3.8.0, and the following versions of the Python packages: numpy==1.20.3; pandas==1.3.5; openpyxl==3.1.2.<br>We used R 4.3.1, and the following versions of the R packages: data.table_1.14.8, cowplot_1.1.1, tidycensus_1.4.4, latex2exp_0.9.6, gridExtra_2.3, zoo_1.8-12, stringr_1.4.0, ggrepel_0.9.1, ggplot2_3.3.5, reshape2_1.4.4, readxl_1.3.1, dplyr_1.0.7, sf_1.0-14

For manuscripts utilizing custom algorithms or software that are central to the research but not yet described in published literature, software must be made available to editors and reviewers. We strongly encourage code deposition in a community repository (e.g. GitHub). See the Nature Portfolio guidelines for submitting code & software for further information.

Data

Policy information about availability of data

All manuscripts must include a data availability statement. This statement should provide the following information, where applicable:

- Accession codes, unique identifiers, or web links for publicly available datasets
- A description of any restrictions on data availability
- For clinical datasets or third party data, please ensure that the statement adheres to our policy

All the data used in the economic module are publicly available and can be obtained as described in the Supplementary Information.
ACS API: https://cran.r-project.org/web/packages/acs/index.html.
BLS: https://www.bls.gov/oes/special.requests/oesm19in4.zip, https://www.bls.gov/cex/2019/msas/norteast.pdf, https://www.bls.gov/webapps/legacy/cpswktab3.htm, https://www.bls.gov/cex/2019/combined/age.pdf,https://www.bls.gov/cex/tables.htm, https://www.bls.gov/cex/pce-concordance-2017.xlsx, https://www.bls.gov/cew/data.htm
BEA: https://www.bea.gov/industry/input-output-accounts-data, https://apps.bea.gov/itable/iTable.cfm?ReqID=70&step=1&acrdn=5, https://apps.bea.gov/industry/xls/underlying-estimates/PCEBridge 1997-2019 SUM.xlsx

The mobility data used in the epidemic module are available from Cuebiq, available upon request submitted to https://www.cuebiq.com/about/data-for-good/.
The Foursquare data are available from the public Foursquare API:https://foursquare.com/products/places (Accessed 16-02-2021). https://developer.foursquare.com/docs/build-withfoursquare/categories/ (Accessed: 09-12-2020).

Human research participants

Policy information about studies involving human research participants and Sex and Gender in Research.

Reporting on sex and gender	We do not report on sex and gender of the participants.
Population characteristics	See below.
Recruitment	See below.
Ethics oversight	The privacy-enhanced mobility data was collected by the company Cuebiq using anonymized records of GPS locations from users that opted-in to share the data anonymously through a General Data Protection Regulation (GDPR) and California Consumer Privacy Act (CCPA) compliant framework. Additionally, we obtained IRB exemption to use the mobility data from the MIT IRB office. (COUHES protocol #1812635935 and its extension #E-2962)

Note that full information on the approval of the study protocol must also be provided in the manuscript.

Field-specific reporting

Please select the one below that is the best fit for your research. If you are not sure, read the appropriate sections before making your selection.

☐ Life sciences ☒ Behavioural & social sciences ☐ Ecological, evolutionary & environmental sciences

For a reference copy of the document with all sections, see nature.com/documents/nr-reporting-summary-flat.pdf

Behavioural & social sciences study design

All studies must disclose on these points even when the disclosure is negative.

Study description	In this study real-world data have been used to initialize the attributes of individual agents in the agent-based simulation. In particular, mobility data have been used to initialize the contacts in the community and workplace layers of the epidemic module. Tabular census data have been used to initialize the features of synthetic individuals and national accounting data have been used to initialize the economic input-output structure.
Research sample	The main agents of the epidemic-economic ABM are the 416,442 individuals of a synthetic population that is representative of the NY MSA. The agents are heterogeneous in several of their socio-economic characteristics (Figure \ref{fig:figureMaM}B), including age, income, employment status, occupation, possibility to work from home, and the census tract where they live. Individuals are grouped into 153,547 households, whose composition is consistent with census microdata. We derive socio-economic characteristics of synthetic individuals from tables provided by the American Community Survey (ACS) and the Bureau of Labor Statistics (BLS), trying to get as many joint distributions of variables as we can. More details on the synthetic population building algorithm and validation tests can be found in the Supplementary Information.<br><br>In the economic module, we also treat industries as agents, considering a single representative firm per industry. We use the 2-digit NAICS level of aggregation, giving 20 different industries. Industries are mainly dependent on one another through the input-output network of consumption of intermediate goods. Since no official data for the NY MSA exist, we downloaded national data from the

	Bureau of Economic Analysis (BEA) and then used a regionalization method to obtain an input-output table that distinguishes between the NY MSA and the Rest of the US.
Sampling strategy	The synthetic population size is determined by the availability of mobility data, collected from the geo-locations of 316070 anonymous opted-in devices collected by the company Cuebiq in the New York Metro Area. Post-stratification techniques has been used to correct for potential biases in the sample of users and to ensure population representation (see Supp. Materials 3.1.1). In particular, because the complete sample of users is slightly biased towards higher income individuals, we downsampled the original sample of users in the Cuebiq dataset to get a more representative distribution of the different quartiles of income. Specifically, the original sample contained 438,178 users and was biased toward high-income people. The penetration rate of high income people was 4.13\% while we only get 2.3\% of people in low-income areas. Thus we downsample the high-income groups to get a more balanced distribution of users by quantile groups. In particular we selected a random sample of 2.3\% of people in each of the income groups. This led us to a final set of 316,070 users. This does not correspond to the size of the synthetic population, as we need to include other agents that are not included in the Cuebiq data. For instance, we need to include children (for ethical and privacy reasons, we cannot access data for individuals less than 18 years old). We obtain the final population as described in Supplementary Sections S3.3.1 and S3.3.6.<br><br>There was no sampling involved for the industry agents - the entire US economy is represented in our model.
Data collection	The main source for the synthetic population is the American Community Survey (ACS). We used the R API that can be downloaded here: https://cran.r-project.org/web/packages/acs/index.html.<br><br>We then collected several labor and consumption data from the Bureau of Labor Statistics (BLS).<br>-income by occupation and industry (https://www.bls.gov/oes/special.requests/oesm19in4.zip)<br>-income by age (https://www.bls.gov/webapps/legacy/cpswktab3.htm, https://www.bls.gov/cex/2019/combined/age.pdf,)<br>-consumption across different categories (https://www.bls.gov/cex/2019/msas/norteast.pdf)<br>-consumption by age and income (https://www.bls.gov/cex/tables.htm)<br>-concordance between BLS and BEA consumption categories (https://www.bls.gov/cex/pce-concordance-2017.xlsx)<br>-Quarterly Census of Employment and Wages (https://www.bls.gov/cew/data.htm)<br><br>We finally collected data on input-output tables and the New York metro area regional economy from the Bureau of Economic Analysis (BEA):<br>-input-output data (https://www.bea.gov/industry/input-output-accounts-data).<br>-regional accounts (https://apps.bea.gov/itable/iTable.cfm?ReqID=70&step=1&acrdn=5)<br>-bridge between BEA consumption and production accounts (https://apps.bea.gov/industry/xls/underlying-estimates/PCEBridge 1997-2019 SUM.xlsx)<br><br>Data collection on mobility was done by the company Cuebiq, rfom the geo-locations of anonymous opted-in mobile phone devices .<br><br>We collected the Foursquare data from the public API: https://foursquare.com/products/places (Accessed 16-02-2021).
Timing	Mobility data were collected from February 17, 2020 to June 30, 2020. The other data are from year 2019, to represent the pre-pandemic situation as accurately as possible.
Data exclusions	We only retain users that we could identify for the full period, i.e. that were observed in the last two months of the period (May-June 2020) at least once, as in Aleta et al., PNAS, 2022 (Ref [16] in the main text). This leads us to the sample containing 438,178 users mentioned above, which is then reduced to 316,070 users for representativeness reasons.
Non-participation	N/A: we did not solicit participation to this study
Randomization	N/A: this is not an experimental/empirical paper with an experimental and control group.

Reporting for specific materials, systems and methods

We require information from authors about some types of materials, experimental systems and methods used in many studies. Here, indicate whether each material, system or method listed is relevant to your study. If you are not sure if a list item applies to your research, read the appropriate section before selecting a response.

Materials & experimental systems

n/a	Involved in the study	
☒	☐	Antibodies
☒	☐	Eukaryotic cell lines
☒	☐	Palaeontology and archaeology
☒	☐	Animals and other organisms
☒	☐	Clinical data
☒	☐	Dual use research of concern

Methods

n/a	Involved in the study	
☒	☐	ChIP-seq
☒	☐	Flow cytometry
☒	☐	MRI-based neuroimaging
